# Polymorphic eruption of pregnancy successfully treated with dupilumab in the postpartum period: A case report

**DOI:** 10.1177/2050313X261446055

**Published:** 2026-05-27

**Authors:** Alexa Moschella, Stephannie Dresden Glockler-Lauf, Niloufar Hosseini, Sophia Colantonio

**Affiliations:** 1 Faculty of Medicine, University of Ottawa, ON, Canada; 2 Division of Dermatology, Department of Medicine, The Ottawa Hospital, ON, Canada; 3 Department of Pathology and Laboratory Medicine, The Ottawa Hospital, ON, Canada

**Keywords:** polymorphic eruption of pregnancy, dupilumab, pruritus, biologic

## Abstract

Polymorphic eruption of pregnancy predominantly occurs during the last month of pregnancy or the immediate postpartum period. Dupilumab, an IgG4 monoclonal antibody, has been utilized in pregnancy and lactation. A 34-year-old gravida 2 para 1 female, on postoperative day 2 following a Cesarean section, presented with pruritic erythematous urticoid papules coalescing into plaques on the abdomen, bilateral arms, legs, and feet, suggestive of polymorphic eruption of pregnancy. A punch biopsy ruled out gestational pemphigoid. Prednisone was contraindicated given her gestational diabetes and gestational hypertension. Dupilumab was administered with betamethasone valerate ointment followed by transition to roflumilast cream. At 6-week follow-up, the skin was cleared of the rash. This case highlights a new potential utility for dupilumab in polymorphic eruption of pregnancy and may represent an alternative to oral steroids in severe or refractory disease.

## Introduction

Approximately 1 in 200 pregnancies are affected by polymorphic eruption of pregnancy, previously known as pruritic urticarial papules and plaques of pregnancy.^1,2^ PEP predominantly occurs in primiparous women during the last month of pregnancy, or in the immediate postpartum period.^1–3^ Clinically, PEP presents with erythematous and edematous papules which may coalesce to form larger urticoid plaques.^2^ They initially arise within abdominal striae and later spread to the trunk, buttocks, extremities, and chest but rarely the face, palms, soles, or mucosa.^2^ Additional polymorphic features, including widespread erythema, eczematous patches, and targetoid lesions may develop.^2^ Although the condition is generally self-limiting, and lesions typically self-resolve over 4–6 weeks, patients often report intense pruritus that significantly impacts their quality of life.^3,4^ Systemic corticosteroids have been utilized for severe and intractable pruritus; however, steroid use in pregnancy confers risks including adrenal insufficiency, fetal anatomical abnormalities, and fetal growth restriction.^5–7^ Dupilumab, an IgG4 monoclonal antibody, has been utilized safely in pregnancy, and without complication during breastfeeding.^8–11^ Therefore, dupilumab may represent a potential alternative to steroid use in severe or refractory disease. Herein, we present the case of a 34-year-old female with severe PEP successfully treated with dupilumab.

## Case report

**Figure 1.**
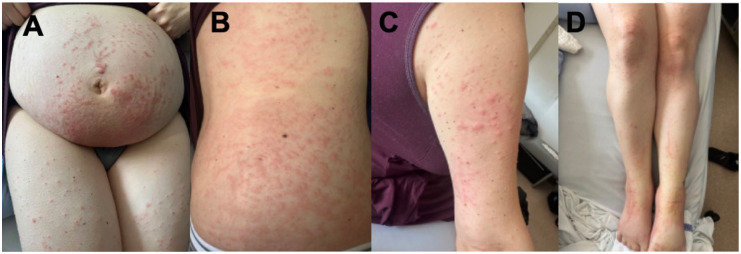
Scattered erythematous urticoid papules coalescing into 2–3 cm plaques on an erythematous base on the abdomen and bilateral upper thighs (a), right lateral trunk (b), right upper arm (c), and bilateral legs (d).

**Figure 2.**
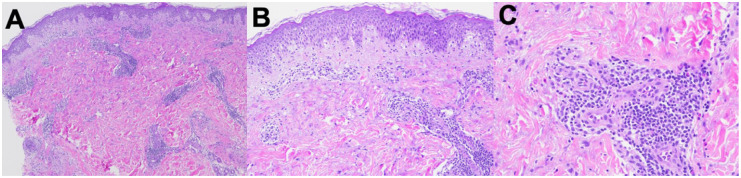
Superficial and mid-dermal inflammatory infiltrate (magnification ×40 (a)); epidermal spongiosis and papillary dermal edema (magnification ×100 (b)); and the inflammatory infiltrate consists of lymphocytes and eosinophils (magnification ×200 (c)).

**Figure 3.**
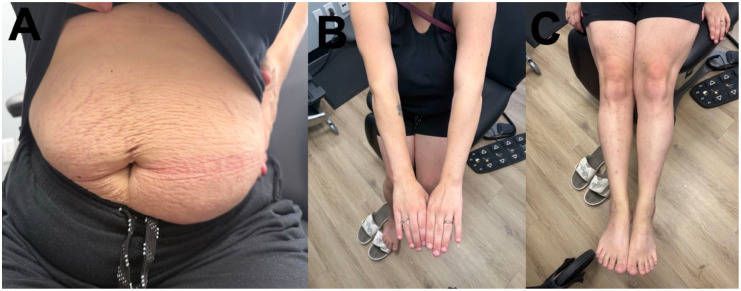
No evidence of rash on the abdomen, (a), bilateral arms (b), with mild xerosis on the bilateral legs (c).

A 34-year-old gravida 2 para 1 (T1P0A1L1) female, on postoperative day 2 following a Cesarean section, reported an intensely pruritic rash that began 2 days prior to delivery. The rash began on the abdomen, and progressed to the back, thighs, lower legs, feet, and arms. She had previously used topical betamethasone valerate 0.1% cream twice daily for 2 days with no effect. Oral loratadine 10 mg tablet (taken once) did not improve pruritus. Past medical history included guttate psoriasis which occurred a few years prior and resolved with roflumilast 0.3% cream, gestational diabetes (treated with insulin) and gestational hypertension (treated with labetalol) during this pregnancy. On examination, small scattered erythematous urticoid papules coalescing into 2–3 cm plaques on an erythematous base were appreciated on the abdomen, bilateral arms, bilateral upper and lower legs, bilateral dorsal feet, and bilateral soles (body surface area (BSA) 15%, Investigator Global Assessment (IGA) 4; Figure 1). Linear erosions were noted on the dorsal feet bilaterally, in keeping with excoriations. Hemoglobin was mildly low (101 g/L normal: 115–155 g/L) although white blood cell count, eosinophils, lymphocytes, and neutrophils were normal. A punch biopsy was obtained from the left upper arm to rule out gestational pemphigoid, which ultimately demonstrated epidermal spongiosis and papillary dermal edema, accompanied by a superficial and mid-dermal perivascular inflammatory infiltrate composed of lymphocytes and eosinophils. The deeper dermis was uninvolved. No evidence of a subepidermal blister or eosinophilic tagging along the basement membrane was identified (Figure 2). Direct immunofluorescence was negative. Given the morphology of pruritic urticoid papules with onset shortly before delivery and worsening thereafter, a diagnosis of PEP was made. Intense pruritus and widespread distribution of the rash warranted systemic treatment. Prednisone was contraindicated given her gestational diabetes and gestational hypertension. We proceeded with dupilumab 600 mg subcutaneously (300 mg administered in right arm and 300 mg administered in right thigh) which was well tolerated with no complications. We also prescribed topical betamethasone valerate 0.1% ointment twice daily to affected areas on the body for up to 3 weeks followed by transition to roflumilast 0.3% cream to affected areas once daily as needed. At 1 week follow-up (11 days postpartum), she reported improvement of her pruritus from 10/10 before dupilumab to 5/10 after dupilumab. She also used betamethasone valerate 0.1% ointment twice daily and over-the-counter moisturizers. On examination, there were erythematous urticoid papules and plaques on the abdomen, legs, and arms, with improvement compared to the previous assessment. Dupilumab 300 mg subcutaneous was administered due to persistence of pruritus, and she continued with her topical management. At 3-week follow-up, her pruritus continued to be rated a 5–6/10, now using topical roflumilast 0.3% cream only. On examination, a few erythematous papules and linear excoriations were noted on the lower legs, while the abdomen and back were clear (BSA 2%, IGA 1). An additional dose of dupilumab 300 mg subcutaneous was administered. At 6-week follow-up, some xerosis remained on the legs; however, the skin was completely cleared of the rash (BSA 0%, IGA 0; Figure 3).

## Discussion

Dupilumab is approved for use in several type 2 inflammatory diseases including atopic dermatitis, prurigo nodularis, asthma, chronic rhinosinusitis with polyposis and eosinophilic esophagitis.^12^ Although type 2 inflammatory diseases have unique clinical presentations, they demonstrate shared pathophysiology, that is, targeted by the novel pharmacology of dupilumab, which disrupts the signaling of interleukin-4 (IL-4) and IL-13, two major drivers of type 2 inflammation.^12^ To our knowledge, this is the first reported case of PEP treated with a dupilumab. Although the pathogenesis of PEP remains elusive, proposed mechanisms include abdominal distension-related damage to underlying collagen connective tissue in the skin triggering an inflammatory response, or a maternal immune reaction to fetal antigens circulating in the bloodstream.^4^ Our patient’s rapid itch and complete response to dupilumab may suggest that type 2-mediated inflammation may play a role in PEP. Overall, our patient was treated with a 600 mg loading dose at week 0, a 300 mg dose at week 1, and a final 300 mg dose at week 3. The significant improvement of pruritus within 1 week of dupilumab injection suggests that dupilumab is an effective treatment for PEP and offers another treatment option for patients who have gestational diabetes or gestational hypertension where conventional treatments such as prednisone are contraindicated.

The utility of dupilumab for gestational dermatoses may have vast potential. A recent meta-analysis of pregnant patients with atopic dermatitis found that dupilumab is likely safe during pregnancy, with no significant increase in the risk of miscarriage or congenital malformations compared to the general population.^11^ Dupilumab has also been used to treat gestational pemphigoid, with multiple documented cases demonstrating improvement.^6^ Although data regarding dupilumab use in breastfeeding are limited, uncomplicated breastfeeding courses with dupilumab have been reported.^9,10^ Expert opinions consider dupilumab acceptable during breastfeeding^13–15^ as its large molecular weight may suggest minimal transfer to breast milk.^15,16^

Overall, this case demonstrates therapeutic potential for dupilumab in the treatment of severe PEP. Prior to this case, oral antihistamines and corticosteroids were predominant systemic agents to treat PEP,^2^ highlighting the need for more systemic treatments including dupilumab. Further studies are required to investigate the longitudinal safety and efficacy of dupilumab in the treatment of PEP in pregnancy and postpartum periods.
